# Individual Topographic Variability Is Inherent to Cortical Physiology but Task-Related Differences May Be Noise

**DOI:** 10.1371/journal.pone.0128343

**Published:** 2015-05-26

**Authors:** Luis F. H. Basile, João R. Sato, Henrique A. Pasquini, Mirna D. Lozano, Mariana P. Nucci, Bruna Velasques, Pedro Ribeiro, Renato T. Ramos, Renato Anghina

**Affiliations:** 1 Laboratory of Psychophysiology, Faculdade da Saúde, UMESP, São Paulo, SP, Brazil; 2 Division of Neurosurgery, Department of Neurology, University of São Paulo Medical School, São Paulo, SP, Brazil; 3 Center of Mathematics, Computation and Cognition, Federal University of ABC, Santo André, SP, Brazil; 4 Department of Radiology, University of São Paulo Medical School, São Paulo, SP, Brazil; 5 Department of Psychiatry, Federal University of Rio de Janeiro, Rio de Janeiro, RJ, Brazil; 6 Department of Psychiatry, University of São Paulo Medical School, São Paulo, SP, Brazil; 7 Department of Neurology, University of São Paulo Medical School, São Paulo, SP, Brazil; National Scientific and Technical Research Council (CONICET)., ARGENTINA

## Abstract

The observation of highly variable sets of association neocortical areas across individuals, containing the estimated generators of Slow Potentials (SPs) and beta oscillations, lead to the persistence in individual analyses. This brought to notice an unexpected within individual topographic similarity between task conditions, despite our original interest in task-related differences. A recent related work explored the quantification of the similarity in beta topography between largely differing tasks. In this article, we used Independent Component Analysis (ICA) for the decomposition of beta activity from a visual attention task, and compared it with quiet resting, recorded by 128-channel EEG in 62 subjects. We statistically tested whether each ICA component obtained in one condition could be explained by a linear regression model based on the topographic patterns from the other condition, in each individual. Results were coherent with the previous report, showing a high topographic similarity between conditions. From an average of 12 beta component maps obtained for each task, over 80% were satisfactorily explained by the complementary task. Once more, the component maps including those considered unexplained, putatively “task-specific”, had their scalp distribution and estimated cortical sources highly variable across subjects. These findings are discussed along with other studies based on individual data and the present fMRI results, reinforcing the increasingly accepted view that individual variability in sets of active neocortical association areas is not noise, but intrinsic to cortical physiology. Actual ‘noise’, mainly stemming from group “brain averaging” and the emphasis on statistical differences as opposed to similarities, may explain the overall hardship in replication of the vast literature on supposed task-specific forms of activity, and the ever inconclusive status of a universal functional mapping of cortical association areas. A new hypothesis, that individuals may use the same idiosyncratic sets of areas, at least by their fraction of activity in the sub-delta and beta range, in various non-sensory-motor forms of conscious activities, is a corollary of the discussed variability.

## Introduction

In the last decade, the constant observation of high inter-individual variability in the cortical distribution of various forms of non-sensory-motor electrical activity, has justified the preservation of individual data for group analysis and development of within individual forms of analysis [1–7]. At first, simple tabulation of the cortical areas containing modeled current density foci of averaged Slow Potentials (SPs, [3]), or tabulation of scored foci of activity of SPs obtained in three tasks [4], suggested an impossibility of universal functional mapping of association cortical areas. Later, the possibility that complex results could have depended on the complex tasks used led to the design of simple attention tasks, where the analysis included the generators of SPs and of induced peak-averaged beta oscillations [5–7], and more recently, of non-averaged ICA decomposed beta activity obtained during the simple visual attention and a reasoning task [1]. In all those cases, individually idiosyncratic topographies were observed, both of averaged SPs or beta, and non-averaged beta ICA patterns, as well as their modeled current density generators. This robust individual variability, in the set of cortical areas presenting various forms of activity, valid for the non-sensory-motor domain, is an increasingly accepted fact, corroborated by other methods such as fMRI and PET, whenever individual data are presented [2,8–15]. It is also corroborated by the common sense ground of neurosurgeons, familiar with the lack of simple relations between focal lesions and symptomatology, and resulted in the need of individually based approaches in functional neurosurgery [16,17]. Accordingly, in neuropsychological research, the lack of a simple one-to-one mapping between lesion localization and clinical symptoms has led some authors to propose different approaches, [18], applicable to functional studies as well, and in a few cases an explicit advice against group spatial averaging was proposed [15,19,20]. In our view, the fact that most groups, both in EEG/MEG and fMRI research, maintain the use of spatial grand averaging (collapsing sensor montages across subjects or mapping results into an “average brain”, respectively), is the main reason for no widespread recognition of this variability as possibly inherent to neocortical physiology.

The main purpose of our current research, following the establishment of inter-individual variability, is to show the high topographical similarity, within individuals, in non-sensory-motor forms of electrical activity between tasks. This similarity was noticed by visual inspection in a study exactly attempting to distinguish SP generators obtained in three visual tasks [4]. In spite the fact that the statistical analysis depicted a few cortical areas distinguishing the tasks, our interpretation already included the possibility of a statistical fluke, and that those differences could vanish by an increasing sample, for instance. In another studyintending to compare peak-averaged beta cortical topography between a simple visual attention task with the shift to the auditory modality [7], simple methods for analyzing both similarities and differences across tasks corroborated the visually observed lack of major differences between tasks, and the minor differences detected were absolutely unsystematic across subjects, additional evidence for the existence of a common set of areas related to visual or auditory attention. This method was improved in a more recent study [1], for showing the topographic similarity between the same visual attention task and a largely different, reasoning task. A linear regression modeling was used in an attempt to explain up to ten beta ICA patterns obtained in each task by the patterns from the complementary task. Results confirmed the major similarity between tasks, and again, the residual unsatisfactorily explained components were not topographically systematic across subjects. The combined inter-individual variability with task similarity within individuals suggested a new functional localization hypothesis: in ça major part of the non-sensory-motor domain, individuals appear to present an idiosyncratic set of active cortical areas, which do not significantly change across tasks.

In the present study, we intended to improve the method in some respects, and compare exhaustively decomposed beta topography between task engagement (in the standard visual attention paradigm) and quiet resting (with common visual stimulation), by usinga larger sample (n = 62), an improved ICA algorithm for comprehensively extracting beta patterns with better artifact removal by computing 30 components, and the linear regression modeling for quantifying the similarity across experimental conditions. A secondary but interesting aspect of the present work is that we included the results from a small subset of the subjects (n = 7; part of an ongoing separate project), who performed the visual attention task in a fMRI acquisition paradigm, to verify the inter-individual variability in a different measure and by an independent method.

## Materials and Methods

### Subjects

Sixty two healthy individuals with normal vision and hearing, 41 male and 21 female, participated in the study. They ranged in age between 19 and 66 years, with no history of drug or alcohol abuse, and no current drug treatment. All subjects signed consent forms specific to this study, and this entire study was specifically approved by the Ethics Committee of the University (Ethics Committee of Universidade Metodista). Seven of the participants also performed the attention task as an fMRI paradigm.

### Stimuli and Task

A commercial computer program (Stim, Neurosoft Inc.) controlled all aspects of the tasks. Visual stimuli composing the cue-target pairs (S1-S2) of the attention task consisted of small rectangles (eccentricity ±0.8°, S1: 100 ms duration, S2: 33 ms; white background). In half of the trials, the S2 rectangle contained a grey circle—the task target—with ±0.3° of eccentricity. A masking stimulus had the same grey level as the target (a ‘checkerboard’ grey and white square composed by one-by-one pixel size squares), and was continuously present, along with the fixation point, except during S1 and S2 presentation. S1 was followed by S2, with onsets separated in time by 1.6 seconds. The ITI was variable, ranging from 2.3 to 5 seconds. We instructed the subjects that a rectangle would be presented to indicate that 1.6 seconds later it would flash again but quickly, containing or not the target circle. The subject decided whether there was a target inside the S2 rectangle, and indicated presence of the target by pressing the right button with the right thumb or absence of the target by pressing the left button with the left thumb. We explicitly deemphasized reaction time in the instructions and measured performance by the percent correct trials, from the total of 96 trials comprising the task. An eye fixation dot was continually present on the center of the screen, as well as a stimulus-masking background, to prevent after-images. During the resting condition, subjects were required to relax, with eyes opened, with fixation on the same dot as in the attention task, and to ignore the stimuli (same as in the attention task). Half of the subjects (out of which some were regular meditators, n = 18, part of another independent study) were also instructed to attempt to concentrate attention on breathing.

### EEG Recording and acquisition of MRIs

We used a fast Ag/AgCl electrode positioning system consisting of an extended 10–20 system, in a 128-channel montage (Quik-Cell, Compumedics Limited), and an impedance-reducing saline solution which restricted the need for skin abrasion to the reference and ground electrode regions. Impedances usually remained below 5 kOhms, and unstable channels were eliminated from the analysis. To know the actual scalp sampling or distribution of electrodes in each individual with respect to the nervous system, we used a digitizer (Polhemus) to record actual electrode positions with respect to each subject’s fiduciary points: nasion and preauricular points. After co-registration with individual MRIs, the recorded coordinates were used for realistic 3D mapping onto MRI segmented skin models, and later used to set up the source reconstruction equations (distances between each electrode and each dipole supporting point). Two bipolar channels, out of the total 132-channels were used for recording both horizontal (HEOG) and vertical electro-oculograms (VEOG). Left mastoid served as reference only for data collection (common average reference was used for source modeling) and a frontal midline electrode was used as the ground. We used 128-channel DC amplifiers (Synamps 2, Neuroscan- Compumedics) for data collection and the Scan 4.5 software package for initial data processing. The filter settings for acquisition were from DC to 200 Hz, and the digitization rate was 1000 Hz. The EEG was collected continuously, and task-related epochs spanned the interval from 300 ms before S1 to 700 ms after S2 in the visual attention task, the same interval also being used for the resting condition. Baseline was defined from the first 300 ms of either type of epochs. Epoch elimination was performed visually for eye movements and muscle artifacts, and then automatically: visual inspection served to eliminate epochs containing other artifacts spread to many electrodes, such as head/cable movements. Isolated electrodes presenting frequent transient electronic noise were also eliminated visually. Eye blinks were removed from the continuous EEG recordings by PCA filtering, prior to the computation of epochs. We used PCA for this purpose because eye-blinks are spatially stable, and our software performs this individual-specific cleaning in a simple and straightforward way: two or three first PCA components of a short time window explaining a blink define a filter to be applied to the whole continuous data.

Structural MRIs were obtained by a 1.5 Tesla GE machine, model Horizon LX. Image sets consisted of 124 T1-weighed saggital images of 256 by 256 pixels, spaced by 1.5 mm. Acquisition parameters were: standard echo sequence, 3D, fast SPGE, two excitations, RT = 6.6 ms, ET = 1.6 ms, flip angle of 15 degrees, F.O.V = 26 x 26 cm. Total acquisition time was around 8 minutes.

To obtain functional MRIs, seven participants were scanned with a Phillips Achieva 3.0T fMRI scanner (Eindhoven, Netherlands) equipped with 80mT/m gradients and a 8 channel head coil. Head movement was minimized using foam padding and a tape across the foreheads of the subjects. The attention task was presented using E-Prime software version 1.0 (Psychology Software Tools, Inc., Pittsburgh, PA). The fMRI acquisition was based on gradient recalled echo planar images (EPI) for the whole brain. Image parameters: TR = 2s, TE = 30ms, 3mm isotropic voxels, 41 AC-PC oriented slices, FOV = 240x240 mm and matrix 80x80. A total of 354 volumes were collected in 11min8sec.

We collected 3D T1 1mm isotropic voxels (TR:7s; TE:3.2s; t = 5:58s; matrix size: 240x240; FOV:240x240 mm; flip angle = 8 and reconstruction 240 serving as anatomical reference during the registration process. The fMRI data analysis was performed within FMRIB Software Library—FSL version 6.0 (Centre for Functional MRI of Brain—FMRIB, Analysis Group, Oxford, UK, HTTP://www.fmrib.ox.ac.uk/fsl/). The individual activation maps were obtained using pre-statistic processing with motion correction using MCFLIRT [21], slice-timing correction using Fourier-space time-series phase-shifting (regular up), non-brain removal using BET [22], spatial smoothing using a Gaussian kernel of FWHM 5 mm, highpass temporal filtering (Gaussian-weighted least-squares straight line fitting, with sigma = 100.0s). Time-series statistical analysis was carried out using FILM with local autocorrelation correction [23], considering the signal change during the 1.6 sec S1-S2 interval with respect to the variable intertribal interval, with Z (Gaussianised T/F) statistic images were thresholded using clusters determined by Z>2.3 and a (corrected) cluster significance threshold of P = 0.05 [24]. The registration was applied to the high-resolution individual structural images.

### Independent Component Analysis

Original EEG epochs were filtered between 17 and 30 Hz: Butterworth, 96dB roll-off. The resulting filtered epochs were then concatenated and subjected to a fastica algorithm implemented by our group in the R platform. A pilot testing determined that 30 independent components were an adequate number, to include all brain origin componentes, and to give enough room for components explaining artifacts, mainly muscular, since the data are filtered in a single band. Components representing muscular activity (sharp polarity reversals, with voltage extrema comprising immediately neighboring frontal, temporal or occipital electrodes) were typically obvious to visual inspection and not included in the analysis. Besides such obvious cases to visual inspection (as performed by other groups, e.g., [25,26]), suspected muscular components (with voltage extrema spreading a little beyond immediately neighbor electrodes), were subjected to single or double (in some cases few) dipole modeling, and resulting sources localized the liquor in the MRIs determined the elimination of their corresponding ICA maps from further analysis.

Although not the focus of this work, we additionally computed conventional averaged event-related potentials using the same EEG epochs, to verify and illustrate the presence of forms of activity expected to be common across subjects, in the same data set used for the main analysis, where the high topographical variability was expected for the beta range. In this case, we kept the epochs only low-pass filtered (0 to 30 Hz), and performed ICA on the averages to extract the main event-related components.

### Statistics of ICA results

For each subject, a multiple linear regression was independently modeled for each of the ICA channel coefficients of one condition (as the response variable), considering the ICA coefficients of the other condition as predictor variables. Components of suspected muscular or electronic artifactual origin were not considered in the analysis. The basic idea of this analysis is to evaluate whether the two tasks have spatially similar components (or linear combinations between them). We considered components highly similar between conditions, i.e., statistically explained by the complementary task, when the resulting adjusted R2 values of the multiple regression were above the cutoff value of 0.8. In other words, we considered that a component was similar between the two tasks if 80% (or above) of its variance (across channels) could be explained using the components of the other task. The percentage of similar components for each task and subject were tabulated for the global consideration of results.

### Intracranial source reconstruction

The independent components from each condition that eventually would be found not to be explained by the complementary condition were subjected to source reconstruction, to test for a possible common origin across subjects, that is, if engagement in the attention task would be associated with activity in some area common to subjects, not present during resting, and vice versa. MRI sets were linearly interpolated to create 3-dimensional images, and semi-automatic algorithms based on pixel intensity bands served to reconstruct the various tissues of interest. A Boundary Element Model (BEM) of the head compartments was implemented, by triangulation of collections of points supported by the skin, skull and cerebrospinal fluid (internal skull) surfaces. Mean triangle edge lengths for the BEM surfaces were, respectively, 8, 7 and 5 mm. Fixed conductivities were attributed to the regions enclosed by those surfaces, respectively, 0.33, 0.0042 and 0.33 S/m. Finally, a reconstructed brain surface, with mean triangle side of 2 mm, served as the model for dipole positions. Individually measured electrode positions were used, and finely adjusted onto the skin’s surface modeled from the MRIs (2 mm mean triangle side). The analysis program (“Curry 6”, Neurosoft Inc.) then calculated the lead field matrix that represents the coefficients of the set of equations which translate the data space (SNR values in the set of channels per time point) into the model space (the thousands of dipole supporting points). The source reconstruction method itself was sLORETA, with data Lp norm = 2, also part of the Curry6 software package.

## Results

### Task Performance and ICA results

All subjects reported that performance was relatively easy during the attention task, provided that they were strongly attending during the critical time of S2 presentation. The overall average performance in the attention task was 90.2% correct responses (standard deviation 13.2%).

After bad electrode elimination, the remaining electrode sets averaged 113 ± 8 in number. The computation of 30 independent induced beta components from all data sets allowed an increased freedom for artifact elimination as compared to our previous study, and proved sufficient for an exhaustive analysis in this band. The decomposition resulted in an overall number of 12 ± 4 good, typical cortically originated topographic patterns across subjects. There was no significant difference in this number between the attention task and resting, neither between genders, instructions (simple resting versus attention to breathing) or meditation experience.

Visual inspection of ICA maps in most cases, from most subjects, allowed an easy matching between conditions. Some maps, however, appeared similar to two, more rarely three maps, of the corresponding condition. This appears to indicate that ICA decomposes the beta range into mostly different, but in a minority of cases, into overlapping spatial patterns. The current density reconstruction results, described below, confirms this impression, where most maps correspond to separate sources in the form of one to a few cortical patches, but sometimes two (or three) maps correspond to largely or partly overlapping sources.

**Fig 1:**
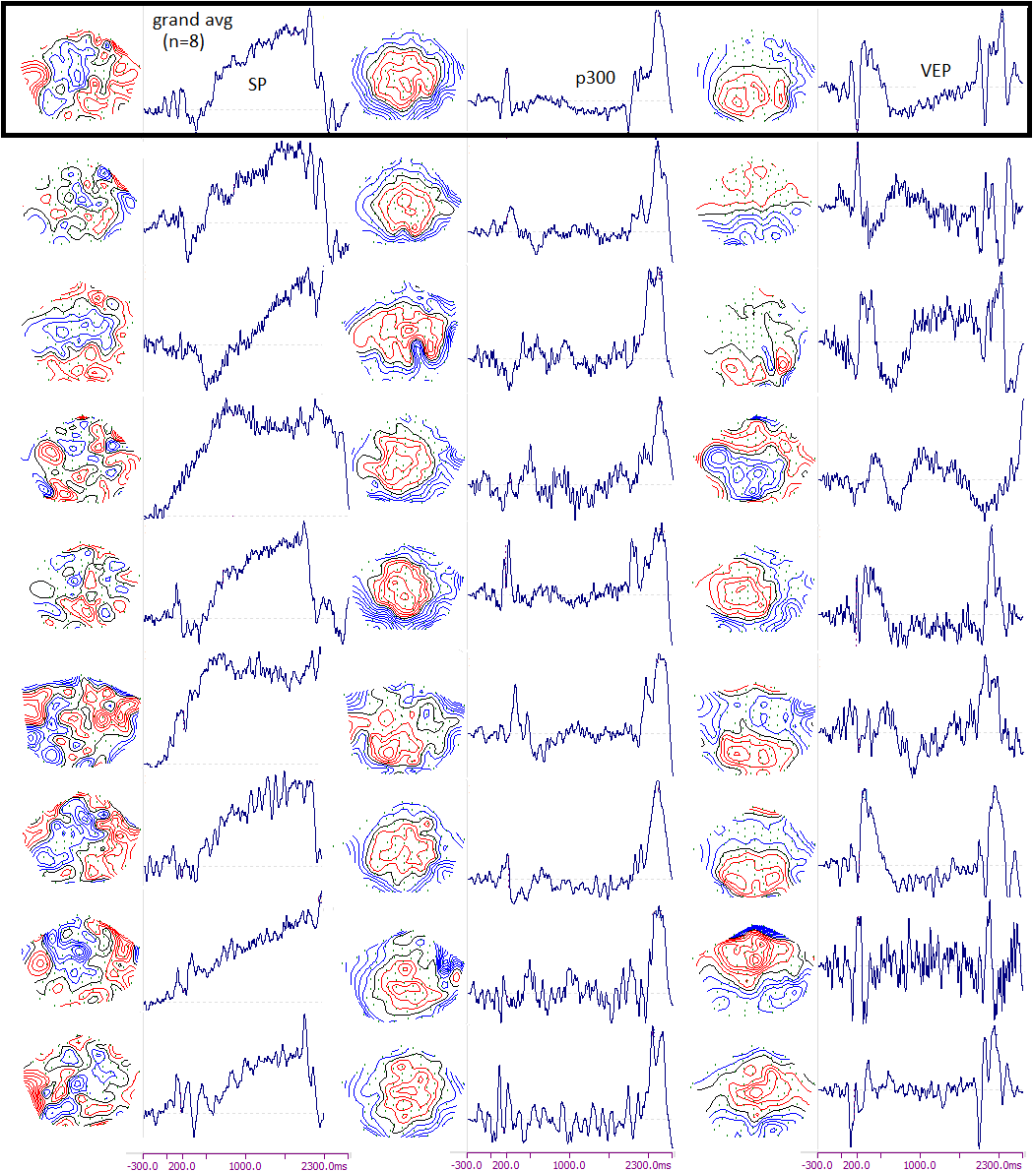
Examples of event-related potentials from the attention task. The top line represents the grand average collapsed across the montage of the 8 example subjects (represented by each line below the top). Each of the main components, SPs, VEPs and P300, were extracted by ICA decomposition of the averages, and shown with the topographical pattern and corresponding time course at the right side of the map.

Regarding the conventional event-related potentials, all subjects presented the expected components, including the visual evoked potentials (VEP), small and large P300-class potentials (to the less and more task-relevant, S1 and S2 stimuli, respectively) and Slow Potentials between S1 and S2. Present but less adequate to observe in this data set are motor potentials in the attention task, due to overlap with robust Slow Potentials, and with evoked potentials and the very strong decision-related P300 to S2. Fig 1 shows the topographic maps extracted by ICA of the averages of 8 randomly chosen individuals, along with the grand average computed across the ‘common’ montage of those subjects: in this case, the number of common good electrodes was reduced to 94. This was done only to illustrate examples of more or less adequate grand averaging in EEG, since it involves a reduction of quality of individual information from our main analysis (the actual positions of electrodes with respect to individual nervous systems was recorded, and clearly shows how same electrodes of the montage do not approach the same cortical structures). All subjects presented similar time courses of those three components, more clearly for the P300 and less so for the SPs. The grand average topography is representative for the P300, also to some extent for the evoked potentials, and not representative for the SPs. The modeling of generators of all averaged components goes much beyond the scope of the present work, but we performed current density reconstruction in a few examples, and confirmed at least some common sources across subjects: occipital sources, typically extrastriate and asymmetrical, of the VEPs, and middle temporal cortical sources of the P300, sometimes in addition to more variable parietal or other neocortical sources. The SPs had a complex, multifocal, highly variable set of cortical sources across subjects.

### Statistics of ICA results

**Fig 2:**
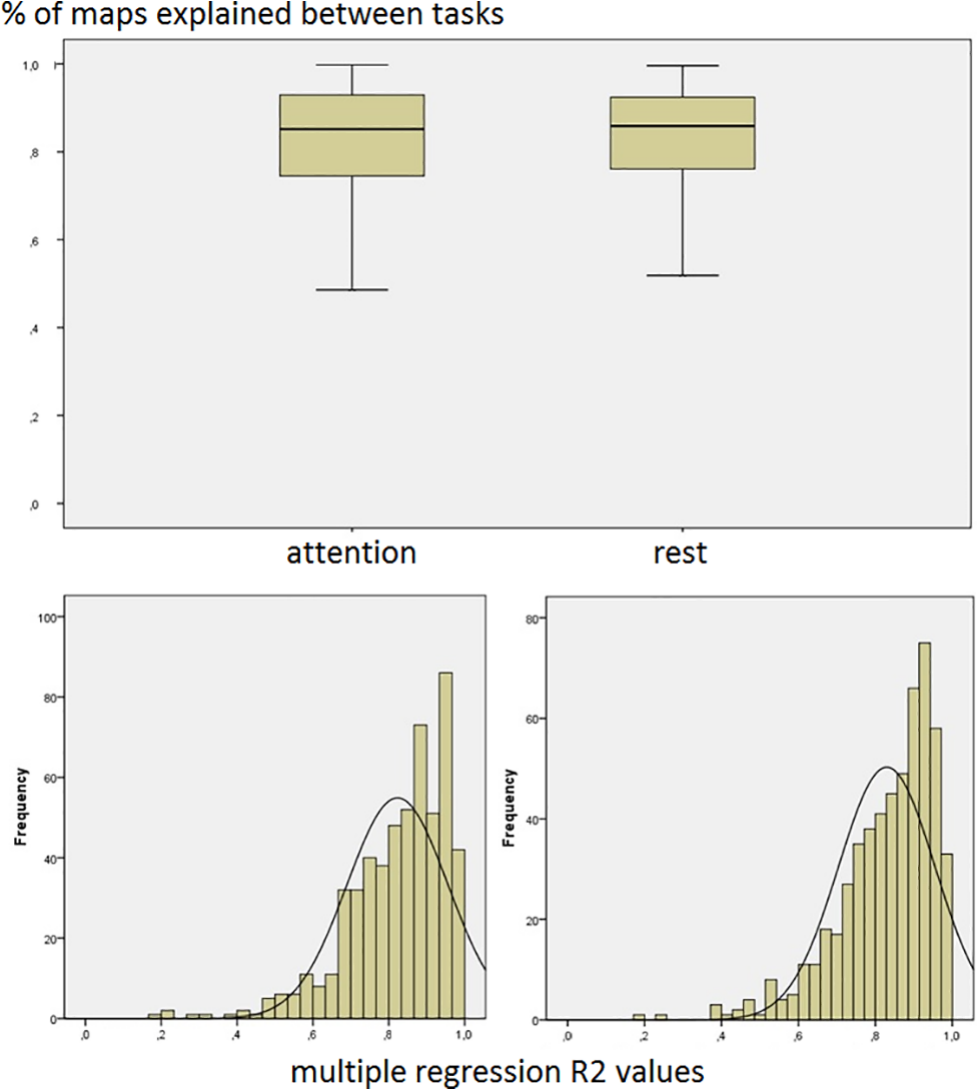
Percentage of explained ICA maps. Top: Average percentage of maps reciprocally explained between the attention task (left) and resting across subjects. Bottom: distribution of linear regression model R2 values across all ICA maps from all subjects, in the attention task (left) and resting (right).

The results from the linear regression modeling agreed with the visual impression, where the overall number of explained components (adjusted R2 above 0.8) was 10 ± 4; when the individual percentages of explained components were considered, the averages were 80% (± 17%) for the attention task and 81% (± 16%) for the resting condition. This difference was not statistically significant (t-test, p = 0.19). Even the overall average values of adjusted R2 across the ‘unexplained’ components across subjects were fairly high, 0.66 (± 0.06) for the attention task, and 0.65 (± 0.10) for the resting condition, indicating similarity even in those arbitrarily cutoff cases rather than clear and important task differences. Thirteen subjects presented all components from the attention task explainable by the resting components, and eleven subjects presented all components from resting explainable by the attention task components. Fig 2 shows the distribution of R2 values obtained from all subjects and both task conditions (777 maps from the attention task and 780 from resting). It also shows the average and 95% confidence interval of the percentage of explained components across subjects in both conditions. One may notice the clear deviation from normality of the R2 values distribution, with a clear concentration above the cutoff value of 0.8.

### CDR results and fMRI BOLD effect during the attention task performance

**Fig 3:**
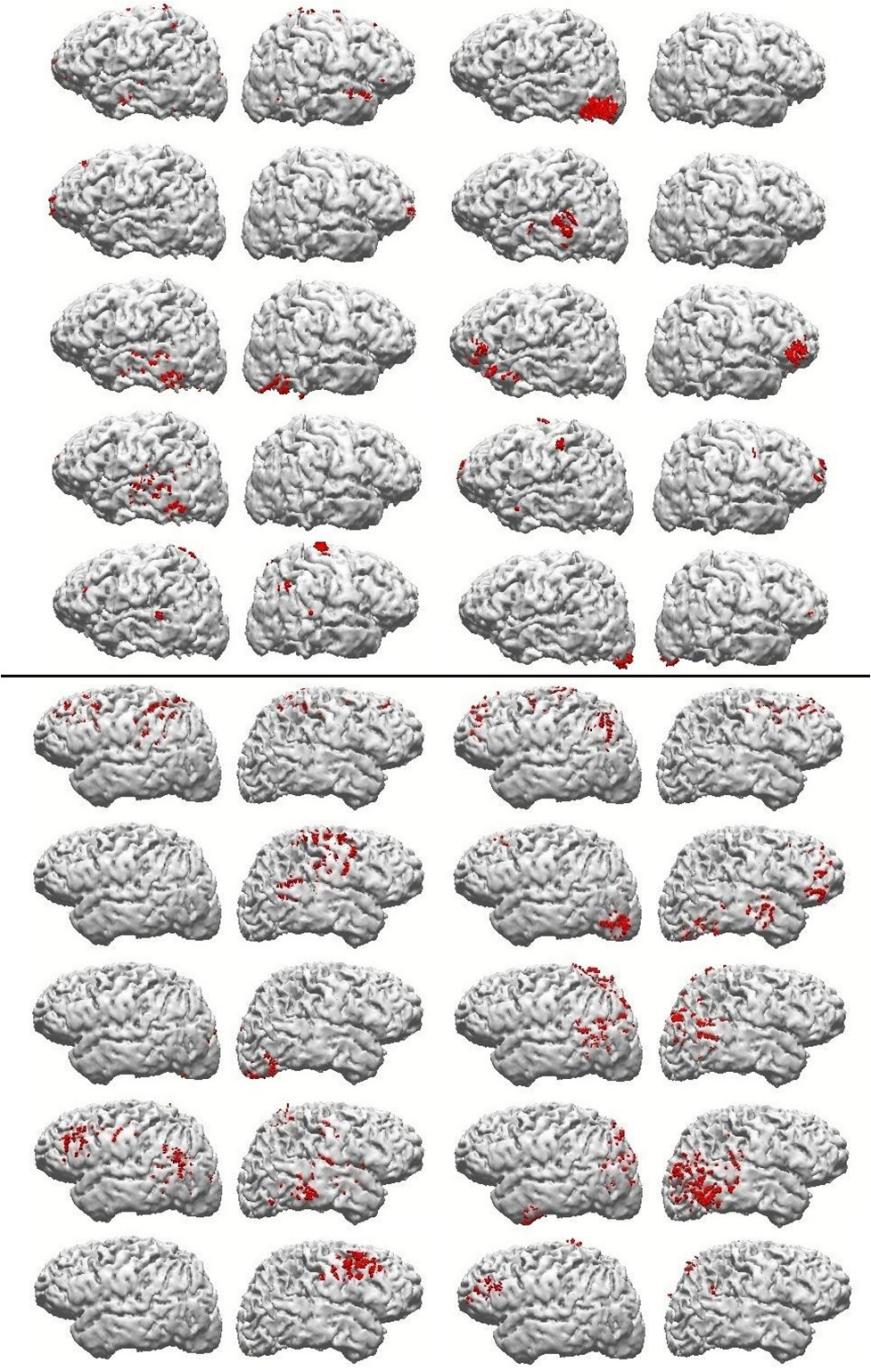
Source reconstruction results. Examples of source reconstruction results obtained for the attention task beta activity, from the two subjects who presented 10 ICA maps explaining beta (sLORETA algorithm, data-Lp norm = 2; current density distribution clipped at the percentile 50 of the maximum current in each case and subject).

**Fig 4:**
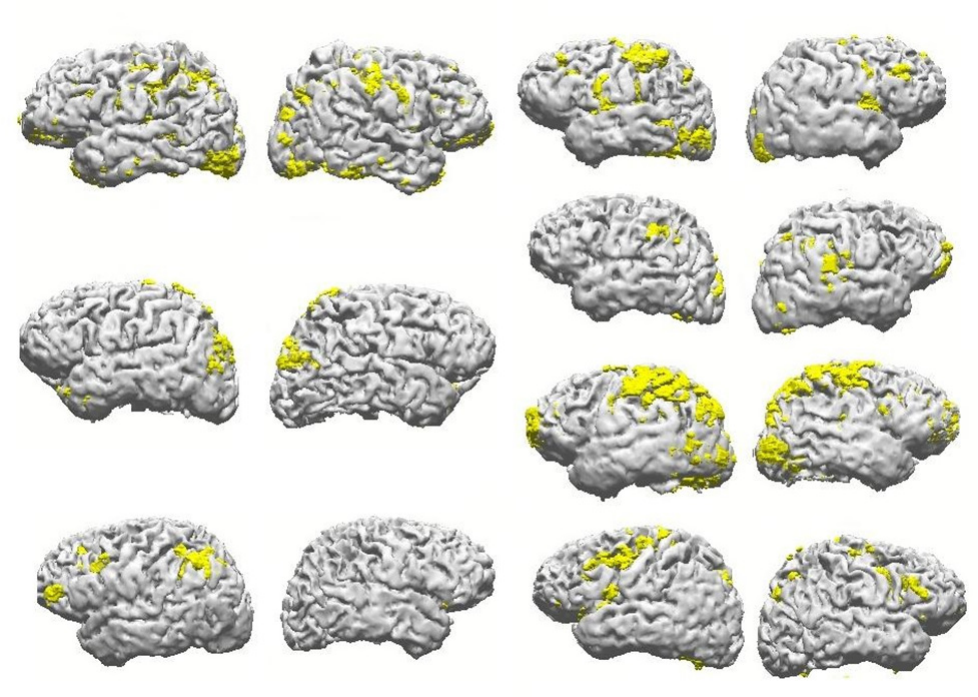
fMRI results. 3D BOLD activation brain images of 7 subjects in individual analysis of the attention task fMRI results. Yellow color represents activation (mean BOLD effect) of the FSL software analysis (p<0.01 to cluster and p< 0.05 to voxel).

The inspection of the CDR results from the few first subjects was once more sufficient to verify the lack of common cortical areas containing beta generators across subjects. Fig 3 shows two example subjects that, in spite of the large number of modeled components (they presented close to average, 10 good components), present clearly different patterns of beta generating areas. Accordingly, the seven subjects presented in Fig 4 are also enough to show the high individual variability in areas presenting the BOLD effect, encompassing the S1-S2 interval of 1.6 seconds, during the attention task. One may notice how no single estimated Brodmann area presenting the effect is common to all subjects. The comparisons between fMRI and CDR results show no obvious overlap of BOLD with beta sources, in spite of the large number of components to attempt this overlap. However, some very preliminary tests suggested the possibility of a good overlap with Slow Potential generators, obtained from the same EEG data. However, this possibility has to be systematically tested in the future, since it will require the development of an independent, specific method for cleaning low frequency artifacts.

### Source reconstruction and cortical distribution of unexplained components

**Fig 5:**
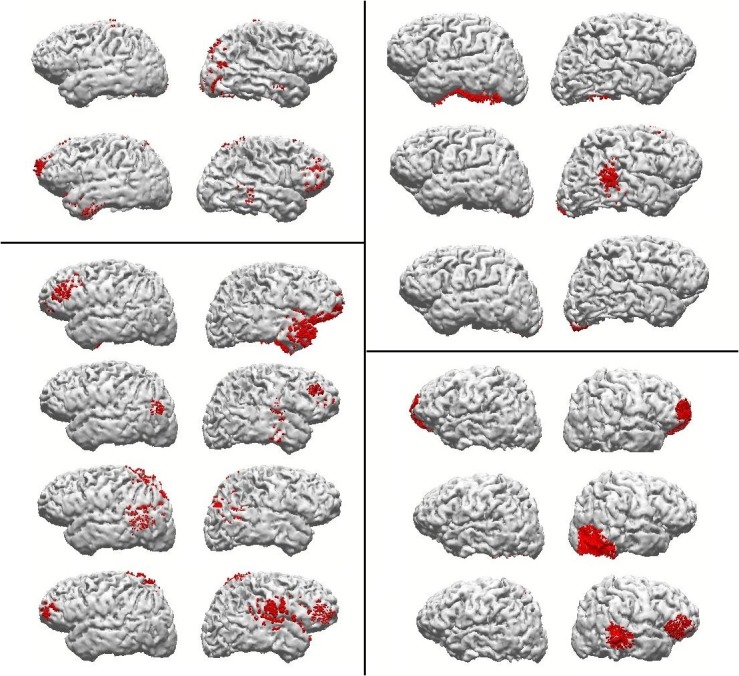
Examples of unexplained reconstruction results. Representation of source reconstruction results (as in Fig 3), of attention task components from four subjects, that were not satisfactorily explained by the resting condition.

In the same manner as the majority of component maps from each individual, those well explained by the complementary condition, the fraction of maps considered ill explained had their estimated generators distributed over cortical areas, as if randomically across individuals. Fig 5 shows examples of reconstruction results for components that were not satisfactorily explained by the complementary task, from four randomly chosen subjects, presenting two, three or four unexplained maps of the attention task. Those few examples are also sufficient to demonstrate the lack of similarity across subjects on what could be a ‘task-specific’ set of cortical areas related to putative additional processes involved in task engagement, as opposed to resting.

## Discussion

### Individual variability is inherent to neocortical association areas function

(“Indvidual variability is not noise” [2])

The present results represent one more of a series of instances where some form of activity was found to be complex, multifocal and highly variable across individuals in distribution. The first encounter with the high inter-individual variability in the distribution of activity across neocortical association areas occurred as the frustration of our original purpose to functionally (universally across subjects) map prefrontal areas with respect to various visual domains (of attention and memory). The studies consisted in the estimation of generators of SPs obtained during visual verbal, pictorial and spatial tasks and in anticipation to supposedly more affectively relevant performance feedback stimuli [3,4]. All subjects were expected to present prefrontal activity in particular areas, according to the anatomy of cortico-cortical connections [27–30] and the supposed frontal generation of SPs (of the Contingent Negative Variation class, CNV; [31,32]). But results showed complex, multifocal foci of current density comprising varying cortical areas, frontal and posterior, highly idiosyncratic to individuals. Since those tasks were complex, and individual strategies were a possible explanation for variability, in the following studies the simplest type of attention task was used, a simplification of Posner’s task [33,34], in which both the location and time of possible targets were known, the same one used in the present study. SPs obtained during this task were equally variable across subjects, with a topographically meaningless grand average, computed by collapsing individual data across the electrode montage (i.e., close in appearance to no single subject; [5]). That same study illustrated the fact that the topography of the visual N200, computed from the same data, in spite of the feeble stimuli used, was typical and clearly similar across subjects. The averaged event-related potentials computed on the present data also lead to the same expected results, regarding topographic similarity or difference across subjects: In spite of the feeble visual stimuli used, all subjects presented the VEPs P100/N200/P200, high amplitude P300-class potentials for the attention task, related to decision making (detection of presence or absence of target in S2), and SPs in the S1-S2 interval. P300 topography was the most similar across subjects, VEPs somewhat variable, possibly due to the feeble visual stimuli used, but preserving the general features of one or two occipital voltage extrema, whereas SPs were highly variable in topography across subjects, with complex multipolar voltage distributions as systematically observed in previous studies. Another putative correlate of attention, the beta rhythm, was studied using the same task, first by peak-averaging the induced beta oscillations [6], and more recently as in the present study, by ICA decomposition of the non-averaged beta activity [1]. In all cases, both scalp voltage distributions and their modeled generators were distributed in a complex, multifocal, highly individually variable fashion. By contrast, both last studiesbrought the opportunity to observe the expected more topographically common forms of activity: By peak averaging, it was demonstrated that the more stimulus-related topography (with evoked potential appearance and thus more similar across subjects) of theta and delta forms of activity were significantly more different between the auditory and visual tasks than beta and Slow Potentials [7]; whereas by direct epoch ICA decomposition, particularly the theta band (not presently analysed), always includes components of obvious sensory evoked topography. By considering the studies (that include not only the attention task) and the relatively infrequent metabolic tracing studies, fMRI and PET, that present individual data [2,8–15], the conclusion was gradually reached that this variability in sets of active neocortical association areas is inherent to (primate) physiology. In particular, this means that such areas cannot have predetermined, universal functions as originally sought. Besides the groups that agree with us that spatial grand averages should be avoided [8–14], some groups explicitly accept this variability as inherent to non-sensorimotor neocortical functioning [2]. It must be made clear at this point, that this recommendation to avoid spatial grand averaging applies only to the non-sensory-motor domain, in situations where not all subjects present activity in any given area. In the opposite case, of experimental conditions when a particular area is the locus of common activity across subjects, for mere localization purposes such as the present case, averaging would not bring useful, additional information to the inspection of individual cases. But whenever subjects need to be compared, such as the interesting case of contrasting a subject with some extreme ability with ordinary healthy individuals [35], an average will be critical for quantification of extent and intensity of task-related physiological changes, and corresponding statistical statements. Spatial averaging can also be useful in studies of modulation of activity by experimental manipulations, in regions of interest defined after the observation of common activity across the sample of subjects (e.g., [36,37]). In principle, spatial averaging will be even more critical whenever activity in areas known to be universally active across subjects, such as the primary cortices or areas demonstrating preferential sensitivity to particular stimulus attributes (as visual motion, color or faces), are the focus of group comparisons, for instance in clinical or pharmacological designs. On the contrary situation typical of the non-sensory-motor domain, spatial averaging will obviously ‘introduce’ activity in the ‘average brain’, in some area where some subjects present no activity at all. One may consider an intermediate situation, of forms of activity akin but not belonging to the sensory-motor domain proper, including for instance error or complex perception-related potentials, or decision making-related potentials, such as the high amplitude P300-class potential observed in the present study, In this case, the use of grand averaging would emphasize the common sources, such as the deep medial temporal cortices generating P300 potentials or extrastriate occipital areas generating the VEPs, and deemphasize possibly variable complementary neocortical sources. Finally, it is interesting to mention here that even some studies on passive stimulation (e.g., cutaneous and nociceptive,[11,12]) explicitly treat the presently discussed type of inter-subject variability, and that some fMRI studies on visual areas with preference to particular stimulus attributes, using a previous localizer scan in each individual, report the lack of response in some subjects, in the cortical area of interest (e.g., [38–40]).

One may now briefly consider the present results on the few individuals that performed the attention task during fMRI recordings. Although small, this preliminary sample size was sufficient to verify no single common area across subjects presenting the BOLD effect. There was, however, no clear spatial overlap with the beta sources, but a preliminary observation of a good overlap with SP components extracted from the same data. Since SPs, comprising most of the power of any EEG recording, are considered the best scalp electrical correlate of the BOLD effect observed in neocortical areas [8–14], we intend, in the immediate future, to better understand the (spatial) relations between the beta-gamma and the SP (sub-delta) ranges of the EEG. This will require the development of a method equivalent to the present one used for the beta range, specifically adapted to separate the distinct low frequency artifacts from the cortical SP sources.

Many authors, however, still believe in particular, universal functions implemented by each cytoarchitectonic association area. The reasons for the discrepancy with related studies that contrast to ours, where apparently common activity across subjects was observed, are discussed in detail in our last work, and include mainly the inadequate *spatial* grand averaging of functional (and anatomical) data, sampling choices, emphasis on differences computed after the loss of individual information, and methodological thresholds [1]. On the other hand, a critical source of support to our hypothesis comes from the practical experience of neurosurgeons and neuropsychologists, familiar with the absolute lack of a one-to-one mapping between lesion localization and clinical symptomatology [18], in particular including the well-known classical cases of major lesions accompanied by none or relatively minor symptoms or incidentally found CT and MRI abnormalities in asymptomatic populations [41–43]. In further agreement with our view is the growing current trend in Functional Neurosurgery, to an individually based approach, with intra-surgical functional mapping beyond verbal eloquent areas [16,17]. Finally, the well acknowledged hardship in inter-laboratory replication of results on the non-sensory-motor domain and the inconclusive status of a universal, final, ‘functional human brain map’, in spite of decades of efforts by so many laboratories, appear to result from an inherent impossibility of such goal. Of course, in principle, the high degree of variability discussed here could be reduced to the form of common patterns to sub-groups of subjects, for instance related to personality traits and proximity of genetic markers. But in our opinion such conclusions may require an unusually and unpractical increase in typical sample size of EEG experiments, due to the complexity of spatial distributions of the variable forms of activity. Equally, other factors such as task difficulty, may in principle individually change the distribution of activity, possibly forcing the use of preferential cortico-cortical pathways and thus approaching subjects in topography. In the present results case, task performance did not significantly correlate with any studied variable, in particular the number of beta components (that keep some proportion to the number of sources) or the percentage of explained components between tasks. Also, those variables did not differ significantly between two sub-groups separated by task performance, and the high variability was qualitatively similar in previous samples using the same task, when the overall performance was lower. This is some indication that the inter-subject variability would not radically change with difficulty, but the proper addressing of the issue will require an intra-individual manipulation of difficulty, as we plan for the immediately following experiments, discussed below.

### Minor task-related differences may be “noise”

Given that the individual idiosyncrasy in sets of active non-sensory-motor neocortical areas, in the high and sub-delta frequency ranges, appears well established, with growing acceptance in the literature, the main point of the present work has shifted to the meaning of task-related physiological differences. Since the beginning of the work on individual data, in spite the original search for differences, visual inspection of the topography of averaged SPs [3–5], peak-averaged beta oscillations [6,7], and more recently non-averaged ICA decomposed beta [1], led to the suspicion that differences were minimal, if at all significant. The tabulation of scored current density (SP modeled generatrors) by individually estimated cytoarchitectonic areas, then subjected to statistical group analysis with preservation of individual anatomical/physiological information, lead to the depiction of a few areas as “task-specific” [4]. But even then, the possibility of a statistical fluke was considered, with results subject to vanish with increasing sample, for instance. The comparison of the P300-class generators obtained in the same study (computed for visual verbal, pictorial and spatial stimuli), following the hypothesis that they represented stimulus detection in the visual ventral versus dorsal pathways [30] or with hemispheric preferences, lead to no significant differences between tasks, in scored activity in any cortical area (unpublished data). In a comparison between the presently used visual attention task and the shift to the auditory modality, simple measures of similarity and differences between the resulting beta generators were computed, and concluded that the differing portion was minimal and non-systematic across subjects [7]. This could be taken as a simple corollary of the inter-individual variability: if no single association area has a fixed, predetermined function, one should expect no area common to subjects implementing the modality shift of attention. In a recent study [1] and in the present one, a more appropriate method to directly measure the topographic similarity between tasks was devised. In both cases, the bulk of the data representing beta activity (the ICA maps) obtained in one task condition were explained by the linear regression model using maps from the complementary condition, indicating largely common topography to all tasks: In the first case, equating in topography the largely differing attention and reasoning tasks, and in the present case, the engagement in the attention task and quiet resting. The instruction to simply rest (and fixate the eyes) versus attend to breathing also lead to no differences in analysed measures. In both studies, the small fraction of individual maps poorly explained by the complementary condition, had their modeled generators once more non-systematically distributed across subjects, as if in a randomic manner.

By taking into account another, critical form of variability, in the moment-by-moment signal changes within individuals [44], much of the claimed task-related physiological differences in the literature appears in need to be revised, especially if resulting from spatial grand averaging of anatomical and physiological data. A future emphasis on within individual forms of analysis could in particular explain the well-known difficulties in replication of non-sensory-motor physiological results across experiments, samples and laboratories.

Our current working hypothesis can be thus summarized: individuals maintain an idiosyncratic distribution of electrically active neocortical association areas, regardless of particular conscious activities performed, when one considers the high (beta and gamma, believed to constitute a physiological *continuum* [45]) and lowest (sub-delta) ranges. Interestingly, those are exactly the EEG frequency ranges best correlated with the BOLD effect in fMRI experiments [46–48]. The high frequencies would represent long-range cortico-cortical communication [49–51], possibly consisting in correlates of a rather general process such as the mere conscious state, instead of attention/arousal or, especially, more circumscribed psychological processes (such as perception, reasoning, etc). This process would be contrasted only with states of sleep or reduced consciousness. SPs, on the other hand, would participate in this general process but also include other forms of cortical activity, such as excitability changes guided by sub-cortical projections, or more local, lateral interactions [52]. According to this hypothesis, other forms of activity, such as the P300-class potentials related to decision making or activity related to error detection and complex perception, mainly expressed in the delta, theta and alpha bands, would represent an intermediate category of processes, between the general, task-unspecific activities and the most individually invariable sensory-motor processes. Their cortical generators would consist in a mixed, partly common set of areas (with a possible prevalence of the philogenetically older medial temporal, cingulate and orbital) in addition to a variable set of neocortical areas. In the immediate following project, we intend to strengthen this working hypothesis by including the comparison between generators (thus using the actual current density distributions, and not restricted to the ICA maps), in a three-way pairwise manner: within-subjects task pairs (with variable difficulty), within-subjects session replication, and between pairs of subjects grouped by anatomical brain similarity (minimal vector distance in elastic transformations of structural MRI data).

## References

[1] Basile LF, Sato JR, Alvarenga MY, Henrique N Jr, Pasquini HA, Alfenas W, et al (2013) Lack of systematic topographic difference between attention and reasoning beta correlates. PLoS One. 2013;8(3):e59595 10.1371/journal.pone.0059595 23544076 PMC3609856

[2] Zilles K1, Amunts K (2013) Individual variability is not noise. Trends Cogn Sci.;17(4):153–5. 10.1016/j.tics.2013.02.003 23507449

[3] Basile LF, Ballester G, Castro CC, Gattaz WF (2002) Multifocal slow potential generators revealed by high-resolution EEG and current density reconstruction. Int. J. Psychophysiol., 45(3):227–240 12208529 10.1016/s0167-8760(02)00014-4

[4] Basile LF, Baldo MV, Castro CC, Gattaz WF (2003) The generators of slow potentials obtained during verbal, pictorial and spatial tasks Int. J. Psychophysiol. **,** 48:55–65 10.1016/s0167-8760(03)00004-7 12694901

[5] Basile LF, Brunetti EP, Pereira JF Jr, Ballester G, Amaro E Jr, Anghinah R, et al (2006) Complex slow potential generators in a simplified attention paradigm. Int J Psychophysiol. 61(2):149–57. 16313987 10.1016/j.ijpsycho.2005.09.004

[6] Basile LF, Anghinah R, Ribeiro P, Ramos RT, Piedade R, Ballester G, et al (2007) Interindividual variability in EEG correlates of attention and limits of functional mapping. Int J Psychophysiol., 65(3):238–51. 17570549 10.1016/j.ijpsycho.2007.05.001

[7] Basile LF, Lozano MD, Alvarenga MY, Pereira JF, Machado S, Velasques B, et al (2010) Minor and unsystematic cortical topographic changes of attention correlates between modalities. PLoS One,5(12):e15022 10.1371/journal.pone.0015022 21179421 PMC3003700

[8] Cohen MS, Kosslyn SM, Breiter HC, Di Girolamo GJ, Thompson WL, Anderson AK, et al (1996) Changes in cortical activity during mental rotation. A mapping study using functional MRI. Brain, 119 (Pt 1): 89–100 8624697 10.1093/brain/119.1.89

[9] Herholz K, Thiel A, Wienhard K, Pietrzyk U, von Stockhausen HM, Karbe H, et al (1996) Individual functional anatomy of verb generation.Neuroimage. 6;3(3 Pt 1):185–94. 9345489 10.1006/nimg.1996.0020

[10] Fink GR, Frackowiak RS, Pietrzyk U, Passingham RE (1997) Multiple nonprimary motor areas in the human cortex. J Neurophysiol; 77(4):2164–74 9114263 10.1152/jn.1997.77.4.2164

[11] Davis KD, Kwan CL, Crawley AP, Mikulis DJ (1998) Functional MRI study of thalamic and cortical activations evoked by cutaneous heat, cold, and tactile stimuli. J Neurophysiol. Sep;80(3):1533–46. 9744957 10.1152/jn.1998.80.3.1533

[12] Hudson AJ (2000) Pain perception and response: central nervous system mechanisms. Can J Neurol Sci. 2;27(1):2–16. 10676581 10.1017/s0317167100051908

[13] Brannen JH, Badie B, Moritz CH, Quigley M, Meyerand ME, Haughton VM (2001) Reliability of functional MR imaging with word-generation tasks for mapping Broca's area. AJNR Am J Neuroradiol; 22(9):1711–8 11673166 PMC7974431

[14] Tzourio-Mazoyer N, Josse G, Crivello F, Mazoyer B (2002) Interindividual variability in the hemispheric organization for speech.Neuroimage;16(3):765–80. 14741679 10.1016/j.neuroimage.2003.08.032

[15] Devlin JT, Poldrack RA (2007) In praise of tedious anatomy. Neuroimage;37(4):1033–41 17870621 10.1016/j.neuroimage.2006.09.055 PMC1986635

[16] Duffau H (2011) The "frontal syndrome" revisited: lessons from electrostimulation mapping studies. Cortex;48(1):120–31. 10.1016/j.cortex.2011.04.029 21621762

[17] Duffau H (2012) A new concept of diffuse (low-grade) glioma surgery. Adv Tech Stand Neurosurg;38:3–27. 10.1007/978-3-7091-0676-1_1 22592409

[18] Noppeney U, Friston KJ, Price CJ (2004) Degenerate neuronal systems sustaining cognitive functions Journal of Anatomy Volume 205 6 15610392 10.1111/j.0021-8782.2004.00343.x PMC1571441

[19] Seghier ML, Friston KJ, Price CJ (2007) Detecting subject-specific activations using fuzzy clustering. Neuroimage;36(3):594–605. 17478103 10.1016/j.neuroimage.2007.03.021 PMC2724061

[20] Mechelli A, Penny WD, Price CJ, Gitelman DR, Friston KJ (2002) Effective connectivity and intersubject variability: using a multisubject network to test differences and commonalities. Neuroimage. 17(3):1459–69. 12414285 10.1006/nimg.2002.1231

[21] Jenkinson M, Bannister P, Brady M, Smith S (2002) Improved optimisation for the robust and accurate linear registration and motion correction of brain images. NeuroImage 17:2(825–841). 12377157 10.1016/s1053-8119(02)91132-8

[22] Smith (2002) Fast Robust Automated Brain Extraction. Human Brain Mapping 17:3(143–155) 12391568 10.1002/hbm.10062 PMC6871816

[23] Woolrich MW, Ripley BD, Brady JM, Smith SM (2001) Temporal Autocorrelation in Univariate Linear Modelling of FMRI Data. NeuroImage 14:6(1370–1386). 11707093 10.1006/nimg.2001.0931

[24] Worsley KJ (2001) Statistical analysis of activation images Ch 14, in Functional MRI: An Introduction to Methods, eds. Jezzard P., Matthews P.M. and Smith S.M.. OUP.

[25] Onton J, Westerfield M, Townsend J, Makeig S (2006) Imaging human EEG dynamics using independent component analysis. Neurosci Biobehav Rev.;30(6):808–22. 16904745 10.1016/j.neubiorev.2006.06.007

[26] Kamiński J, Brzezicka A, Gola M, Wróbel A (2012) β band oscillations engagement in human alertness process. Int J Psychophysiol;85(1):125–8 10.1016/j.ijpsycho.2011.11.006 22155528

[27] Pandya DN, Yeterian EH (1990) Prefrontal cortex in relation to other cortical areas in rhesus monkey: Arquitecture and connections In: Progress in Brain Research, vol.85 Uylings HBM, Van Eden CG, De Bruin JPC, Corner MA, Feenstra MGP. (Eds). Elsevier Science Publishers BV. 63–94. 2094916 10.1016/s0079-6123(08)62676-x

[28] Pandya DN, Seltzer B, Barbas H (1988) Input-output organization of the primate cerebral cortex. Comparative primate biology, 4: 39–80.

[29] Barbas H (1992) Architecture and cortical connections of the prefrontal cortex in the rhesus monkey. Advances in Neurology, 57: 91–115. 1543090

[30] Macko KA, Mishkin M (1985) Metabolic mapping of higher-order visual areas in the monkey. Res. Publ. Assoc. Res. Nerv. Ment. Dis., 63: 73–86 3895333

[31] McCallum WC (1988) Potentials related to expectancy, preparation and motor activity In: Handbook of Electroencephalography and Clinical Neurophysiology. Human Event-Related Potentials (revised series vol.3) Picton TW (Ed.). Elsevier Science Publishers, 427–534.

[32] Fuster JM (1989) The Prefrontal Cortex (2nd ed). Raven Press New York.

[33] Posner MI (1980) Orienting of attention. Q. J. Exp. Psychol. 32 (1), 3–25 7367577 10.1080/00335558008248231

[34] Posner MI, Snyder CR, Davidson BJ (1980) Attention and the detection of signals. J. Exp. Psychol. 109 (2), 160– 174R 7381367

[35] Naito E, Hirose S (2014) Efficient foot motor control by Neymar's brain. Front Hum Neurosci;8:594 10.3389/fnhum.2014.00594 25136312 PMC4118031

[36] Aziz-Zadeh L, Fiebach CJ, Naranayan S, Feldman J, Dodge E, Ivry RB (2008) Modulation of the FFA and PPA by language related to faces and places. Soc Neurosci;3(3–4):229–38. 18979378 10.1080/17470910701414604

[37] Ranganath C, DeGutis J, D'Esposito M (2004) Category-specific modulation of inferior temporal activity during working memory encoding and maintenance. Brain Res Cogn Brain Res;20(1):37–45. 15130587 10.1016/j.cogbrainres.2003.11.017

[38] Xu Y, Turk-Browne NB, Chun MM (2007) Dissociating task performance from fMRI repetition attenuation in ventral visual cortex. J Neurosci;27(22):5981–5. 17537969 10.1523/JNEUROSCI.5527-06.2007 PMC6672266

[39] Haist F1, Lee K, Stiles J (2010) Individuating faces and common objects produces equal responses in putative face-processing areas in the ventral occipitotemporal cortex. Front Hum Neurosci;4:181 10.3389/fnhum.2010.00181 21206532 PMC3009480

[40] Epstein RA1, Higgins JS, Parker W, Aguirre GK, Cooperman S (2006) Cortical correlates of face and scene inversion: a comparison. Neuropsychologia;44(7):1145–58. 16303149 10.1016/j.neuropsychologia.2005.10.009

[41] Eskandary H, Sabba M, Khajehpour F, Eskandari M (2005) Incidental findings in brain computed tomography scans of 3000 head trauma patients. Surg Neurol.;63(6):550–3. 15936382 10.1016/j.surneu.2004.07.049

[42] Katzman GL1, Dagher AP, Patronas NJ (1999) Incidental findings on brain magnetic resonance imaging from 1000 asymptomatic volunteers. JAMA. 7 7;282(1):36–9. 10404909 10.1001/jama.282.1.36

[43] Vernooij MW, Ikram MA, Tanghe HL, Vincent AJ, Hofman A, Krestin GP, Niessen WJ, Breteler MM, van der Lugt A (2007) Incidental findings on brain MRI in the general population. N Engl J Med. 11 1;357(18):1821–8. 17978290 10.1056/NEJMoa070972

[44] Garrett DD1, Samanez-Larkin GR, MacDonald SW, Lindenberger U, McIntosh AR, Grady CL. (2013) Moment-to-moment brain signal variability: a next frontier in human brain mapping? Neurosci Biobehav Rev.;37(4):610–24. 10.1016/j.neubiorev.2013.02.015 23458776 PMC3732213

[45] Llinás RR, Steriade M (2006) Bursting of thalamic neurons and states of vigilance. J Neurophysiol. 95(6):3297–308. 16554502 10.1152/jn.00166.2006

[46] Ekstrom A (2010) How and when the fMRI BOLD signal relates to underlying neural activity: the danger in dissociation. Brain Res Rev.;62(2):233–44. 10.1016/j.brainresrev.2009.12.004 20026191 PMC3546820

[47] Khader P1, Schicke T, Röder B, Rösler F (2008) On the relationship between slow cortical potentials and BOLD signal changes in humans. Int J Psychophysiol.;67(3):252–61. 17669531 10.1016/j.ijpsycho.2007.05.018

[48] Hinterberger T1, Veit R, Strehl U, Trevorrow T, Erb M, Kotchoubey B, et al (2003) Brain areas activated in fMRI during self-regulation of slow cortical potentials (SCPs). Exp Brain Res.;152(1):113–22. 12830347 10.1007/s00221-003-1515-4

[49] Traub RD, Whittington MA, Stanford IM, Jefferys JG (1996) A mechanism for generation of long-range synchronous fast oscillations in the cortex. Nature:383, 621–624. 8857537 10.1038/383621a0

[50] Whittington MA, Traub RD, Kopell N, Ermentrout B, Buhl EH (2000) Inhibition-based rhythms: experimental and mathematical observations on network dynamics. Int J Psychophysiol;38(3):315–36. 11102670 10.1016/s0167-8760(00)00173-2

[51] Bibbig A1, Traub RD, Whittington MA (2002) Long-range synchronization of gamma and beta oscillations and the plasticity of excitatory and inhibitory synapses: a network model. J Neurophysiol.;88(4):1634–54. 12364494 10.1152/jn.2002.88.4.1634

[52] He BJ1, Raichle ME (2009) The fMRI signal, slow cortical potential and consciousness. Trends Cogn Sci.;13(7):302–9. 10.1016/j.tics.2009.04.004 19535283 PMC2855786

