# Correction: Individual Topographic Variability Is Inherent to Cortical Physiology but Task-Related Differences May Be Noise

**DOI:** 10.1371/journal.pone.0131621

**Published:** 2015-06-24

**Authors:** The PLOS ONE Staff

The ninth author’s name is spelled incorrectly. The correct name is: Renato Anghinah. The publisher apologizes for the error.
